# Stretchable Materials for Robust Soft Actuators towards Assistive Wearable Devices

**DOI:** 10.1038/srep34224

**Published:** 2016-09-27

**Authors:** Gunjan Agarwal, Nicolas Besuchet, Basile Audergon, Jamie Paik

**Affiliations:** 1 Ecole Polytechnique Federale de Lausanne (Swiss Federal Institute of Technology, EPFL), Reconfigurable Robotics Laboratory, EPFL-IGM-RRL, MED 11326, Station 9, CH-1015 Lausanne, Switzerland

## Abstract

Soft actuators made from elastomeric active materials can find widespread potential implementation in a variety of applications ranging from assistive wearable technologies targeted at biomedical rehabilitation or assistance with activities of daily living, bioinspired and biomimetic systems, to gripping and manipulating fragile objects, and adaptable locomotion. In this manuscript, we propose a novel two-component soft actuator design and design tool that produces actuators targeted towards these applications with enhanced mechanical performance and manufacturability. Our numerical models developed using the finite element method can predict the actuator behavior at large mechanical strains to allow efficient design iterations for system optimization. Based on two distinctive actuator prototypes’ (linear and bending actuators) experimental results that include free displacement and blocked-forces, we have validated the efficacy of the numerical models. The presented extensive investigation of mechanical performance for soft actuators with varying geometric parameters demonstrates the practical application of the design tool, and the robustness of the actuator hardware design, towards diverse soft robotic systems for a wide set of assistive wearable technologies, including replicating the motion of several parts of the human body.

Due to multiple inherent advantages offered by most soft materials such as light weight, low manufacturing cost, large number of degrees of freedom and high adaptability, soft robotics^1^,^2^,^3^,^4^,^5^,^6^,^7^,^8^,^9^,^10^,^11^,^12^,^13^,^14^,^15^ has emerged as a dynamically evolving field of research in the recent past, combining expertise from diverse fields of engineering including materials science, chemistry and mechanics, to create novel systems with pre-programmed capabilities out of elastomeric materials, capable of withstanding large deformations. Soft robotic systems and structures can potentially be useful for applications in diverse fields, ranging from bioinspired and biomimetic systems^3^,^4^,^5^,^6^, adaptable locomotion on unstructured terrains and autonomous navigation^7^, to gripping and manipulating fragile objects^1^,^6^, operating surgical tools^8^ and biomedical rehabilitation^9^.

Soft actuators are a critical component of soft robotic systems and can achieve actuation by employing a variety of methods, including actuation with the aid of electrical charges^5^,^10^,^11^, chemical reactions^12^, shape-memory alloys^5^,^10^, and pressurized fluids^1^,^3^,^9^. Soft pneumatic actuators (SPA) are particularly attractive for implementation in wearable robotic systems due to their ease of fabrication, safety of operation, high power-to-weight ratio, and low cost^13^,^14^,^15^. These actuators are typically composed of air corridors and chambers made from deformable materials, where the input air pressure is applied.

Depending on the design and structure of the actuator, SPAs can be used to generate a variety of different motion profiles, such as linear extension, contraction, bending and rotary motion, and apply mechanical forces or moments within the desired range. This makes them potentially very useful for practical implementation in human-assistive, wearable devices, targeted at either restoring or replicating the motion of parts of the body, or assisting with activities of daily living. There are several such examples of assistive, rehabilitative and wearable devices in literature which utilize SPAs as the principal actuator to drive them. Figure 1a tabulates some such assistive devices that have been studied, along with a comparison of the performance obtained with a single actuator employed in these devices, in terms of motion and force profiles. This comparative study is used as a benchmark to define the design criterion for developing a new, robust actuator, along with the performance metrics desired from the novel prototype for some of the applications mentioned.

Figure 1b shows schematic diagrams of a couple of assistive soft robotic system prototypes that can be built with such actuators and are capable of delivering desired motion and force profiles for targeted applications in motion assistance and biomedical rehabilitation. In Fig. 1b (left), a SPA-driven assistive exoskeleton shown can enable locomotion rehabilitation in mammals with neurological disorders. SPAs which generate linear extension drive the system in this case and are connected to rigid attachments mounted onto the body to constrain motion in the desired plane. As shown in Fig. 1b (right), the compliance and light weight of SPAs can be exploited to achieve rehabilitation in patients suffering from reduced mobility in their hands through the means of a soft assistive robotic human glove. In this system, bending actuators capable of exhibiting high flexibility and large blocked torque capacities are connected over each finger of the hand and are actuated in parallel to guide and assist hand movement.

Despite the established potential of SPAs and their diverse implementation towards robotic systems for meeting desired functional requirements in crucial applications such as the ones described above, the lack of repeatability in currently existing SPA design and fabrication procedures greatly limits their potential and performance in such systems. Discussed in further detail below, existing SPAs are largely confined to laboratory prototypes and their reported results are difficult to reproduce due to multiple, and in several cases, complex manufacturing steps requiring high manual precision or intervention, and unpredictable, uncontrolled mechanical failure. Because mechanical performance of SPAs depends greatly on the geometric parameters, small changes in the fabrication timing, sequence, or environment can contribute significantly to the actuator performance, making the production of repeatable results very challenging.

In an effort to circumvent some of these fabrication and repeatability issues, newer actuator designs have been developed where the actuators are made in a single molding step^16^,^17^. This is in contrast to the actuators that base their designs in multiple forms of McKibben actuators or pneumatic artificial muscles^18^,^19^,^20^,^21^ with reinforcing windings^9^,^16^,^22^,^23^ or have multiple air chambers with narrow connecting passages^1^,^24^. In one new simple design^16^, the actuator body is fabricated in a single step, and while the fiber reinforcements are still manually wound on the body, grooves guide the uniform placement of fiber. While this procedure ensures repeatability in fiber placement, due to manual winding of fiber reinforcements, the repeatability of the prototype is not guaranteed. To replace fiber structures with a more robust and manufacturable reinforcement, wrapped soft pneumatic artificial muscles (WSPAMs)^17^ employ a thin silk mesh structure to constrain bloating (excessive radial expansion) of the actuator core. Since the mesh structure is glued onto the actuator on all sides, it overconstrains the entire motion range of the actuator and prevents the actuator from delivering large bending angles. While it is possible to somewhat tune the bending angle obtained by modifying the mesh pattern, it is observed that the issue of bloating becomes more pronounced as the wrap pattern is changed to have thinner reinforcements. To address these issues related to the fabrication and performance of the actuators, there is a strong motivation to develop a new design and fabrication procedure for the soft actuators that ensures robustness along with manufacturability.

In parallel with the development of newer designs and manufacturing techniques for SPAs, there has also been an increased interest in the development of predictive models for these actuators in the recent past, since predicting the performance of the actuators is non-trivial due to the non-linear behavior of the materials used, the complex interaction between multiple materials employed and the sophisticated geometries involved. Several studies have employed analytical models to describe the mechanical deformations in SPAs^25^,^26^,^27^ within the desired range. To capture more detailed information on stress-strain distributions within the actuators and model the non-linear effects observed at larger strains more accurately, finite element analysis (FEA) of SPAs has also been carried out in the past for specific materials, geometries, and applications^16^,^22^,^23^,^28^,^29^,^30^. It is worth noting that although a wide variety of non-linear constitutive material models have been developed over the past few decades to describe the mechanical behavior of elastomers, these have not yet been fully utilized to model and predict the large deformations incurred in some soft actuators within the robotics domain. Due to the complexity involved in modeling non-linear behavior exhibited by soft materials under loading, while some of the previous work in this field has focused solely on the linear elastic material response of SPAs at small strains^22^, other work^23^,^28^ has utilized hyperelastic material laws such as the Mooney-Rivlin^31^ and Neo-Hookean^32^ models based on linear approximations of the strain invariants, which yield limited accuracy at higher strains^33^. To address this issue, non-linear soft material behavior in SPAs has been captured across a large range of realistic strains in^16^,^29^,^34^ using more appropriate hyperelastic models. However, the actuators modeled in these studies suffer from lack of manufacturability and repeatability.

In the present study, we present two major findings from the development of a novel actuator design and its experimental results compared to predictions from the FEA based design tool. The presented two-part, shell-reinforced, SPA design allows “fool-proof” prototyping of both bending and linear actuators and produces results in the desired performance range. The presented numerical models using FEA accurately predict the complex mechanical response and the performance obtained with the designed actuators while allowing rapid design iterations to optimize the design parameters. The design tool and models used in the current study are also available open-source on the Reconfigurable Robotic Laboratory website (http://rrl.epfl.ch) where it is possible to use the models developed in the present work as a starting point to modify and create different geometries and materials for any robotic application.

## Results

**Figure 1:**
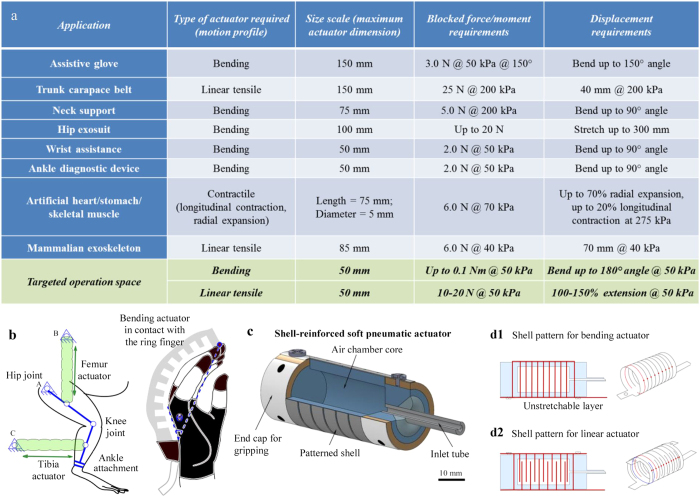
(**a**) A review of design and performance metrics for soft actuators used in some human-assistive, wearable devices listed in literature, along with references. (**b**) Schematic diagrams showing two such assistive devices that employ soft pneumatic actuators, for spinal cord rehabilitation using a soft exoskeleton (left) with linear actuators, and for assisting hand motion using a robotic glove (right) with bending actuators. (**c**) Schematic view of the proposed SPA. The actuator comprises of a soft elastomeric silicone core onto which a shell structure made of a much stiffer material is attached. (d) Schematic showing laser-cut patterns on shell for forming bending (d1) and linear (d2) frames. The bending frame, seen in d1, comprises multiple, equally spaced cuts. The shell is rolled-up as shown, with a thin strip of uncut material forming the unstretchable layer to guide motion in bending. The number of cuts on shell surface is varied to achieve variable stiffness of the structure. The linear frame has a pattern as shown in d2, with alternating slits of the same length. The corresponding shell obtained upon attaching two such symmetric patterns together is seen on the right, to achieve guided linear motion.

The technique used to fabricate the SPA presented in this work ensures robustness and repeatability in performance, since it leaves minimal manual intervention and margin for manual error. The two main parts of the actuators developed here are the actuator body and the un-stretchable shell. The actuator body is made out of highly elastomeric Ecoflex^TM^ 00–30 (Smooth-on-Inc.^TM^, PA, USA). The un-stretchable shell, made from much stiffer polyethylene terephthalate (PET), (Q-Connect^TM^), is mounted onto the actuator body and constrains its motion and mechanical performance in the desired range. A wide variety of materials were tested to select the best combination of materials for forming the actuator. For forming the actuator core, which is required to simultaneously exhibit high flexibility as well as mechanical strength at relatively low input pressure values (up to 50 kPa), highly stretchable materials such as Ecoflex^TM^ 00–10, Ecoflex^TM^ 00–20, Elastosil^TM^ and Dragonskin^TM^ were tested. Actuators formed from Ecoflex^TM^ 00–10 and Ecoflex^TM^ 00–20 were observed to be not as robust (unable to withstand pressures up to 50 kPa without rupture or significant leakage) as those formed from Ecoflex^TM^ 00–30, due to the lower shore hardness for these materials as compared to Ecoflex^TM^ 00–30. On the other hand, actuators formed from Elastosil^TM^ and Dragonskin^TM^ were found to be too stiff and required application of much higher pressures (>80 kPa) to achieve the same deflection as that achieved with Ecoflex^TM^ 00–30. Based on these results, Ecoflex^TM^ 00–30 was selected as the material to form the actuator core for further study and characterization. For forming the actuator shell reinforcement, a variety of materials were tested as well. These included PET, Kapton^TM^, fabric and nylon. Due to fabrication complexities and lack of repeatability issues incurred with some of these materials considered such as fabric and nylon, where the contained elastomer core was not adhering well enough to the reinforcement material to maintain a strong, robust contact and suffered from leakage during elastomer curing, and Kapton^TM^, in which case the shell was more difficult to cut precisely and glue onto the core, PET was chosen as the preferred material to form the shell and considered for further study. Thus, the combination of soft materials used here delivers the desired range of high flexibility as well as adequate robustness in actuation, at the same time. A schematic view of the actuator is shown in Fig. 1c.

The actuator core comprises of a single air chamber, created in a single-step molding process. The un-stretchable shell mounted on top of the actuator body surface constrains the actuator body to inflate in only the desired configuration. The pattern created on the shell surface governs the pattern of displacement obtained with the actuator. Although it is possible to achieve other motion profiles as well by varying the patterns on the shell surface, bending and linear actuators were selected for further study in this work, patterns corresponding to which are shown in Fig. 1d. The number of equally-spaced cuts (thus effectively the width of the uncut portion) on the shell surface was varied for both the types of actuators to study its effect on performance (more details on actuator fabrication and shell design are provided in the Supplementary Information).

**Figure 2:**
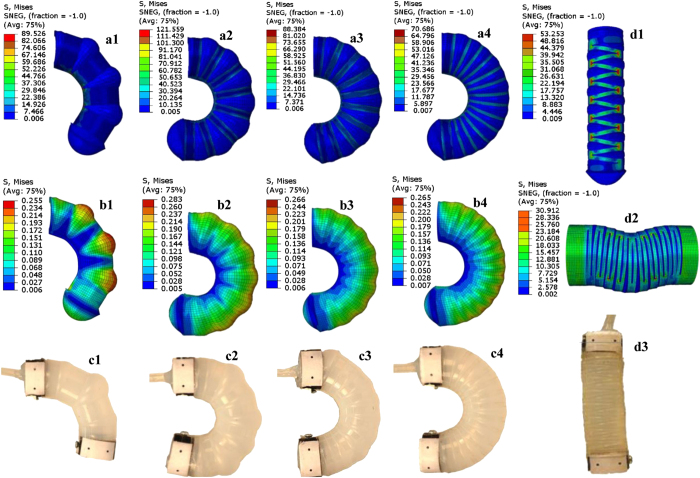
(**a–c**) Simulation vs. experimental results for bending actuators with various geometries, with 3, 7, 9 and 13 cuts on shell surface shown in c1–c4 (from left to right). The top panel (a1–a4) shows the Von Mises stress contour plots for stresses for the entire actuator structure, combining both the shell and the core material, while the bottom panel (b1–b4) shows the stresses in the soft core alone, for the corresponding geometries in the top panel. All stress values are in MPa. Comparing images in (**a**,**b**), it is seen that much larger stresses are incurred in the shell, owing to its significantly larger stiffness (in GPa as compared to the stiffness of the core material in kPa). (**d**) Von Mises stress contour plots for a linear actuator with 13 cuts on shell surface in free displacement testing (d1) and in blocked force testing (d2). An experimental image of the actuator in free displacement testing is shown in d3.

To determine the material properties and characterize the complex mechanical behavior for the stretchable materials used, we conducted thorough mechanical testing (detailed description in the Supplementary Information). Having ascertained the properties of the comprising materials as well as the individual actuator specimens, we developed a methodology for creating numerical simulations of our soft actuated materials. Computational modeling was done by using FEA in ABAQUS/Standard (Simulia, Dassault Systems) to simulate the performance of the actuators and predict the performance of different design iterations of the soft materials used (further details on the FE model are described in the Methods Section). 3-D models were created for both bending and linear actuators, and for several geometries of actuators tested experimentally. Computationally, these tests are modeled using half-symmetry, with the external face containing the air inlet fixed in all directions. Figure 2 shows the Von Mises stress contour plots obtained from the FEM simulations, along with the experimental images of the pressurized actuators at the corresponding values of input pressure.

**Figure 3:**
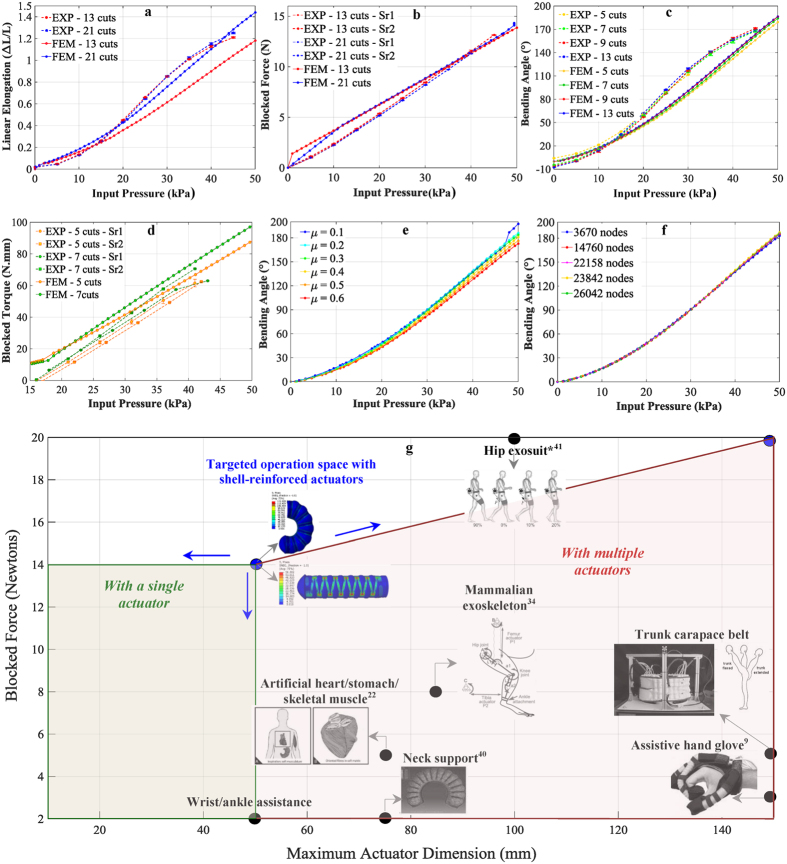
Comparison of simulation and experimental results for linear actuators with 13 and 21 cuts on shell surface, in (**a**) free displacement testing and (**b**) blocked force testing. In general, the simulation results approximate the experimental values well. Comparison of simulation and experimental results for bending actuators with 5, 7, 9 and 13 cuts on shell surface, in (**c**) free displacement testing and (**d**) blocked torque testing. For blocked torque testing, in this case, the actuator is first bent to a certain angle and then clamped in place. (**e**) Simulation results obtained with varying values of coefficient of friction *μ* between shell surface and actuator body, for bending actuators with 9 cuts on shell surface in free displacement condition. (**f**) Results from mesh convergence testing for a bending actuator with 9 cuts on shell surface in free displacement testing. The legend shows the total number of nodes in the system, including both the shell and the actuator surfaces. (**g**) Maximum blocked force obtained at 50 kPa input pressure vs. actuator size scale, for actuators employed for the different assistive, wearable devices and applications listed previously in Fig. 1a, along with targeted performance space achieved with actuators presented here. This includes devices for wrist and ankle assistance, a trunk carapace belt, an assistive hand glove^9^, an artificial heart/stomach/skeletal muscle^22^, a mammalian exoskeleton^34^, neck support^40^, and a hip assist exosuit^41^ (‘*’Indicates a different actuation mechanism, but comparable performance metrics).

Experimental data was gathered for linear actuators undergoing free displacement and blocked force testing and compared to the simulation results. To characterize the actuators, the applied input air pressure is increased in steps from zero up to a maximum value of 45 kPa, in 10–15 s. More details on the experimental testing and characterization of actuators are described in the Methods Section. Figure 3a compares results from experiments and simulations for free displacement obtained as a function of input pressure. Unlike the case for previous generation of SPAs^34^, with testing over 1200 cycles, we observed no noticeable Mullins effect^35^ in these shell-reinforced actuators, signifying a considerable design and repeatability improvement over existing actuators. The simulations replicate the experiments well, with a maximum deviation of up to 16% within the range of pressures considered. Figure 3b compares results from experiments and simulations for maximum blocked force delivered by the linear actuators. It is observed both experimentally and in the simulations that actuators with lower number of cuts on shell surface resist blocked force testing well without buckling at pressures up to 50 kPa, since larger surface area of the air chamber is constrained in this case.

Experimental data is also gathered for bending actuators undergoing free displacement and blocked force testing and compared to the simulation results. Seen previously in Fig. 2a,b, in general, the stress distribution within the actuator becomes more uniform with the increase in the number of cuts on the shell surface. This is as expected since increasing the cuts on the stiffer shell surface should allow for more uniform expansion of the contained highly deformable silicone material. As less (<5% of the chamber diameter before inflation) bloating of air chamber is permitted at higher number of cuts due to larger surface area for expansion at the contact interface, the pressure needed to obtain a given angle also slightly decreases (up to 2 kPa over a total pressure range of 50 kPa) with the increase in number of cuts on shell surface. Thus, the frequently encountered issue of bursting instabilities in such actuators can be mitigated here by changing the geometry of the shell to include larger number of cuts on shell surface. Figure 3c compares results from both experiments and simulations for free displacement obtained as a function of input pressure. Until lower pressures of 15 kPa, the simulations predict larger displacements with a maximum variation up to 7.5%, potentially due to the backlash in the setup. With further increase in pressure, simulation results fall up to a maximum of 16% below the experiments before catching up again at pressures of around 40–50 kPa. Figure 3d compares the results from experiments and simulations for blocked torque tests for the bending actuators. The simulations are seen to replicate the experimental results well, indicating a good fit.

## Discussion

In order to assess the applicability of these actuators to assistive wearable devices discussed previously, we carried out a comparison of the performance delivered by the shell-reinforced actuators to some of the existing actuators. It should be noted that this study is primarily targeted at more efficient design and improved characterization of the soft actuator interface with the body towards assistive wearable devices. Depending on the desired application of focus, the pressurized air reservoirs, pumps, valves and other equipment required for operating these soft actuators would need to be scaled down appropriately, so as to make these devices portable. The actuators developed in the present study do not exhibit any detectable leakage and deliver consistent performance across a wide range of input pressure loads. A scatter plot is shown in Fig. 3g which plots the maximum blocked force obtained at 50 kPa input pressure with actuators employed for the different devices and applications listed previously in Fig. 1a. Applying excessively high input pressures to the actuators in order to generate large magnitudes of forces not only requires more complex and bulkier pneumatic pumping platforms but also may result in mechanical failure and/or reduced functionality of the actuators, making the actuators not as lucrative for assistive, wearable systems, where lightweight, safety and stability is desirable. Thus it is desirable to obtain large, controllable forces and displacements from these systems at low values of input pressures. It is seen from the plot in Fig. 3g that most of listed devices employ actuators that can generate less than 8 N of blocked force at the mentioned value of input pressure. For most of these applications under consideration, for instance in gripping and support, it is preferable to have the capability to generate forces and displacements in a wide range so as to have versatile systems capable of gripping both fragile and tough objects with the same system, or providing support at different levels of mechanical stiffness.

The robust pneumatic actuators presented here help achieve this target. At relatively low input pressures of 40–50 kPa, these actuators are observed to deliver at least twice as much force as compared to other existing actuator prototypes, of approximately 14 N, without introducing any mechanical instability in the actuators or the comprising high-elasticity materials. The mechanical performance can be easily modulated to deliver lower values of force by simply lowering the input pressure or by varying the geometric pattern of the shell wrapped onto the actuator and interchanging the shell. The same flexibility can be achieved with the designed actuators in terms of motion capabilities. At low input pressures, the actuators are capable of generating large values of linear elongation with up to 150% stretch. The bending actuators developed here can easily deliver high bending angles of up to 200° well within the range of the previously mentioned low pressures, making them highly attractive for implementation in applications requiring flexibility such as hand, neck or knee assistance. Combining all of these performance metrics delivered by the actuators, along with the design criterion defined by the assistive devices considered, it is concluded that the actuators presented in the current study hold great potential for revolutionizing the development of soft assistive wearable systems with advanced capabilities.

## Methods

### FE Model of Soft Actuators

To model the highly non-linear mechanical behavior of the actuator body, an appropriate hyperelastic model was used. Uniaxial and planar tests in tension were performed. Further details on material testing are described in the Supplementary Information. Multiple general hyperelastic constitutive laws which may be expected to produce reasonable results for a broad range of materials, such as the Yeoh model^33^, Ogden model^36^, Arruda-Boyce model^37^, polynomial-type models^38^ and Van-der-Waals model^39^ were evaluated. After testing compatibility with the material data, the model ultimately selected for this material was the Ogden model^36^. The final parameters for the 3-term Ogden model are: μ_1_ = 1.887 × 10^−3^, μ_2_ = 2.225 × 10^−2^, μ_3_ = 3.574 × 10^−3^, α_1_ = −3.848, α_2_ = 0.6632, α_3_ = 4.225, D1 = 2.9259, D_2_ = D_3_ = 0. All μ terms are in units of N/mm^2^, all α terms are dimensionless, and all D terms are in units of 1/(N/mm^2^). To model the mechanical behavior of the shell material (PET), a linear elastic model was used, with a Young’s modulus of 2 GPa and a Poisson’s ratio of 0.35. The actuators reach a steady state and exhibit stable mechanical response within a time frame of 1–3 s after the application of internal pressure loading. Since the current study was aimed primarily at characterizing and comparing the performance obtained with different SPA designs in the steady state, the dynamics of airflow into the actuator core was not taken into consideration in the model here. The response of the SPAs to different levels of pressure loading was tested to find quasi-static solutions using the dynamic, implicit solving scheme. As compared to a fully static solution, the quasi-static solution improves the convergence of the model at higher strains. The air pressure load was modeled as an applied boundary condition on the entire internal surface of the core, and ramps up linearly from zero to the desired value in pseudo-time, to study the effect of varying levels of input pressure on the mechanical performance obtained with the SPA. Surface-to-surface contact between the shell elements and the actuator body elements was maintained using the slip interaction property, with finite sliding along the tangential direction. A penalty friction formulation was used to incorporate a finite coefficient of friction with isotropic directionality, with value in the range of 0.1 to 0.6. Figure 3e shows simulation results for free displacement for a bending actuator with different values of friction between the shell and the actuator body. Based on this comparison, a nominal friction coefficient of 0.2 was chosen for both bending and linear actuators simulations. For bending actuators, an additional constraint was imposed in the simulations to accurately replicate the actuator structures. To guide the bending motion, a narrow, 3 mm wide unstretchable layer portion of the shell surface, oriented perpendicular to the length of the slits, and spanning the entire length of the actuator was glued onto the actuator body. In the simulations, a tie constraint was imposed between the shell surface and the actuator body at this specific region to replicate adhesive contact between the two interacting surfaces. The mesh used in the models was refined to include a bias in the regions that are the most stressed during the course of motion. Mesh convergence testing effectively removed all mesh sensitivity from the analysis. The results are plotted in Fig. 3f and show the effect of mesh element size, along with the effect of changing the total number of nodes in the mesh for the entire system, on the bending angles obtained as a function of input air pressure.

### Experimental Characterization of SPAs

We experimentally validated the performance of the SPA prototypes in terms of free displacement for both the bending and linear actuators, blocked force for linear actuators, and blocked torque for bending actuators. For free-displacement experiments, the proximal end-cap with the air inlet was clamped in a rigid fixture. The other end of the actuator was permitted to move freely while inflating, thereby generating a curved or straight trajectory, depending on whether a bending or linear actuator was tested. The experiments were performed three times with each geometric variant to analyze the positioning repeatability as well as the pressure-to-angle repeatability. To test the maximum blocked force delivered by the linear actuators, each end-cap of the actuator was rigidly clamped, with the actuator in an un-pressurized state. On the distal clamped end of the actuator (i.e., the end opposite to the end with the air inlet), a six-axis force/torque sensor was used to measure the force produced by the tip of the actuator as the input pressure was ramped up from zero. The blocked torque testing for bending actuators was done in two steps. First, the actuator was clamped only at the end containing the air inlet, and the unconstrained end was allowed to inflate up to a fixed angle. In the second step, while maintaining the final orientation of the actuator achieved in the first step (by maintaining the input pressure required to achieve the same at a constant value), the free distal end-cap of the actuator was clamped rigidly. The actuator was then subsequently inflated to higher pressures, starting from the level of pressure attained in the course of step 1, and the force/torque sensor was used to measure torque generated at the tip of the actuator.

## Additional Information

**How to cite this article**: Agarwal, G. *et al*. Stretchable Materials for Robust Soft Actuators towards Assistive Wearable Devices. *Sci. Rep.* **6**, 34224; doi: 10.1038/srep34224 (2016).

## Supplementary Material

Supplementary Information
