# Supplementary material for: Stretchable Materials for Robust Soft Actuators towards Assistive Wearable Devices
Source: Sci Rep. 2016 Sep 27;6:34224. doi: 10.1038/srep34224 (PMC5037455; doi:10.1038/srep34224)
Supplement: Supplementary Information [file srep34224-s1.pdf]

Stretchable Materials for Robust Soft Actuators towards Assistive Wearable Devices

Gunjan Agarwal, Nicolas Besuchet, Basile Audergon and Jamie Paik

Supporting Information

I) Material testing and characterization

Hyperelastic material properties are typically determined through simple tests on small material samples, performed across a large range of relevant strains. The primary tests are tension and compression tests in the uniaxial, biaxial, and planar (plane stress) directions. As actuators are not loaded in compression, those tests are not considered here. Additionally, while uniaxial and planar tests can be performed with standard test equipment, biaxial testing requires specialized equipment in order to achieve properly-equilibrated two-dimensional strains. Therefore, only uniaxial and planar tests in tension were performed here. A universal material test system (MTS Criterion Model 42 Load Frame) was used for the material characterization. Dog-bone shaped samples prepared according to the ASTM guidelines were tested in uniaxial tension testing. Multiple samples of Ecoflex^{TM} 00-30 with different dimensions were prepared in a single batch and were tested at strain rates varying from 1 mm/s to 10 mm/s. Images of some samples as-prepared and during testing are shown in **Figure S1.**a and Figure S1.b, along with tabulated dimensions in Figure S1.g.

A typical stress-strain curve for the Ecoflex^{TM} 00-30 material obtained from uniaxial testing is shown in Figure S1.c. Planar testing was also done with rectangular specimens with dimensions of 50 x 50 x 5 mm, which were loaded at a rate of 1%/s up to approximately 275% strain. Stress-strain curves were plotted with the material data obtained for maximum strains of up to 350%. Once the experimental data was acquired, a least-squares fit of the stress-strain equations was computed to determine the parameters of the hyperelastic model. After testing compatibility with the material data, the model ultimately selected for this material was the Ogden model, as described in the Experimental Section. The uniaxial and planar data, along with a 6-term Ogden model fit is shown in Figure S1.d.

In addition, stress relaxation tests were carried out to characterize any time-dependent effects of the material. Since the applications for the actuator are indented for room temperature, the material characterization was also done at room temperature. The tests were carried out by applying a high tensile strain rapidly to a test specimen and held constant

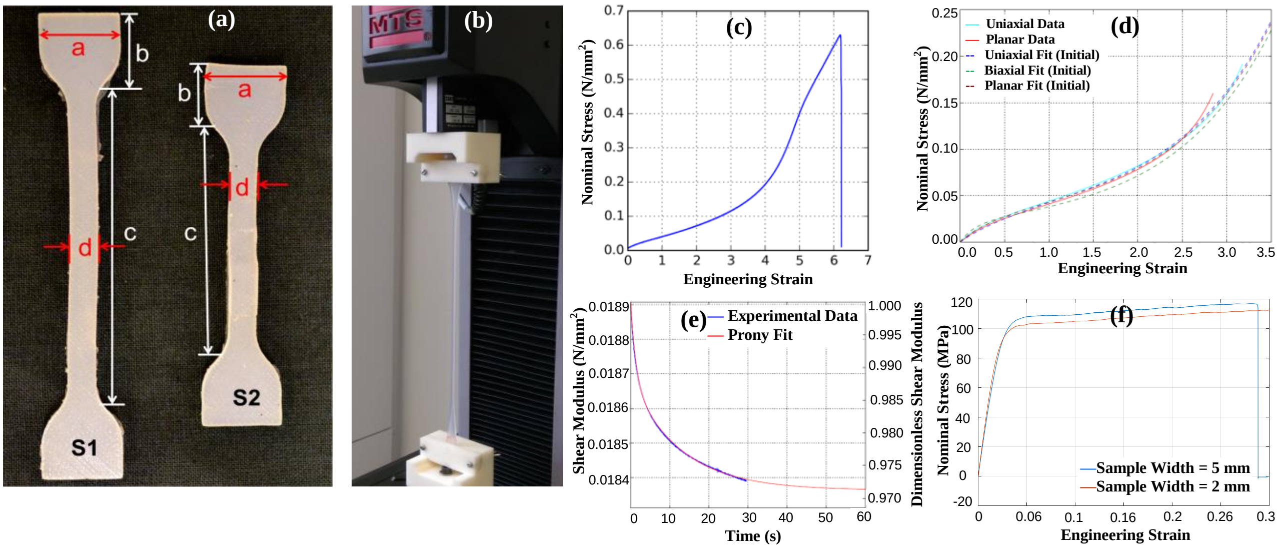

**(g)** Dimensions (mm)	Sample S1	Sample S2
a	15	15
b	15	15
c	65	45
d	5	5
E (thickness)	3	3

Figure S1: Images showing the test samples made from Ecoflex^{TM} 00-30 (a) under uniaxial tensile mechanical testing (b) using a universal material test system (MTS Criterion Model 42 Load Frame). High strains of up to 600% were recorded with the material. (c) Stress-strain curve for Ecoflex^{TM} 00-30 obtained from uniaxial testing. (d) Uniaxial and planar material data for Ecoflex^{TM} 00-30, along with a 6-term Ogden model fit. (e) Prony series fit to stress relaxation testing data for Ecoflex^{TM} 00-30. (f) Stress-strain curve for PET obtained from uniaxial testing. (g) Dimensions of dog-bone shaped samples made from Ecoflex^{TM} 00-30 material for uniaxial tensile testing are reported in the table.

for a period of time, during which stress was measured. After acquiring material data, the decay of stress was fit to the Prony series mathematical model, the results of which are shown in Figure S1.e. It was seen from the dimensionless shear modulus in this plot that the relaxation effects were less than 3%. Thus, the viscoelastic effects were judged to be minimal for the material chosen and were not included further in the model.

To model the mechanical behavior of the shell material (PET), a linear elastic model was used. To obtain material properties including the elastic modulus of the material, the yield stress, and the stress-strain history, samples of the material were tested for uniaxial tensile strength with samples designed according to the ASTM standards, at strain rates varying from 1 mm/s to 10 mm/s. A typical stress-strain curve for PET material obtained from uniaxial testing is shown in Figure S1.f. From uniaxial testing, the elastic modulus of the material was found to be 2 GPa and the yield strength was tested to be 95 MPa. A Poisson's ratio of 0.31 was considered for the material. Since stresses higher than the yield values were never reached in the model, the behaviour of the material is considered to be linear elastic for simplicity.

II) Actuator design and fabrication

The technique used to fabricate the soft pneumatic actuators presented in this work ensures robustness and repeatability in manufacturing, since it leaves minimal manual intervention and margin for error. The actuators presented here are composed of two main parts: 1) the actuator body and 2) the un-stretchable shell. The actuator body is made out of highly elastomeric siloxane material (Ecoflex^{TM} 00-30, Smooth-on-Inc., PA, USA). The un-stretchable shell is made from a much stiffer plastic material (polyethylene terephthalate (PET), Q-Connect^{TM}). **Figure S2.**a shows the images of the two types of fabricated actuators, in bending and linear motion (Figure S2.a.ii and Figure S2.a.iii, respectively).

The actuator comprises of a single air chamber, created in a single-step molding process, details of which are shown in Figure S2.b. As mentioned previously, this is done to avoid the traditionally used two-step molding process for creating soft pneumatic actuators in which two separate halves need to be glued to form the air chambers, thereby reducing fabrication errors and possibility of delamination at the interface. At each end of the chamber, an additional length of rubber material is provided to facilitate attachment of fixtures for gripping the actuator during testing, since

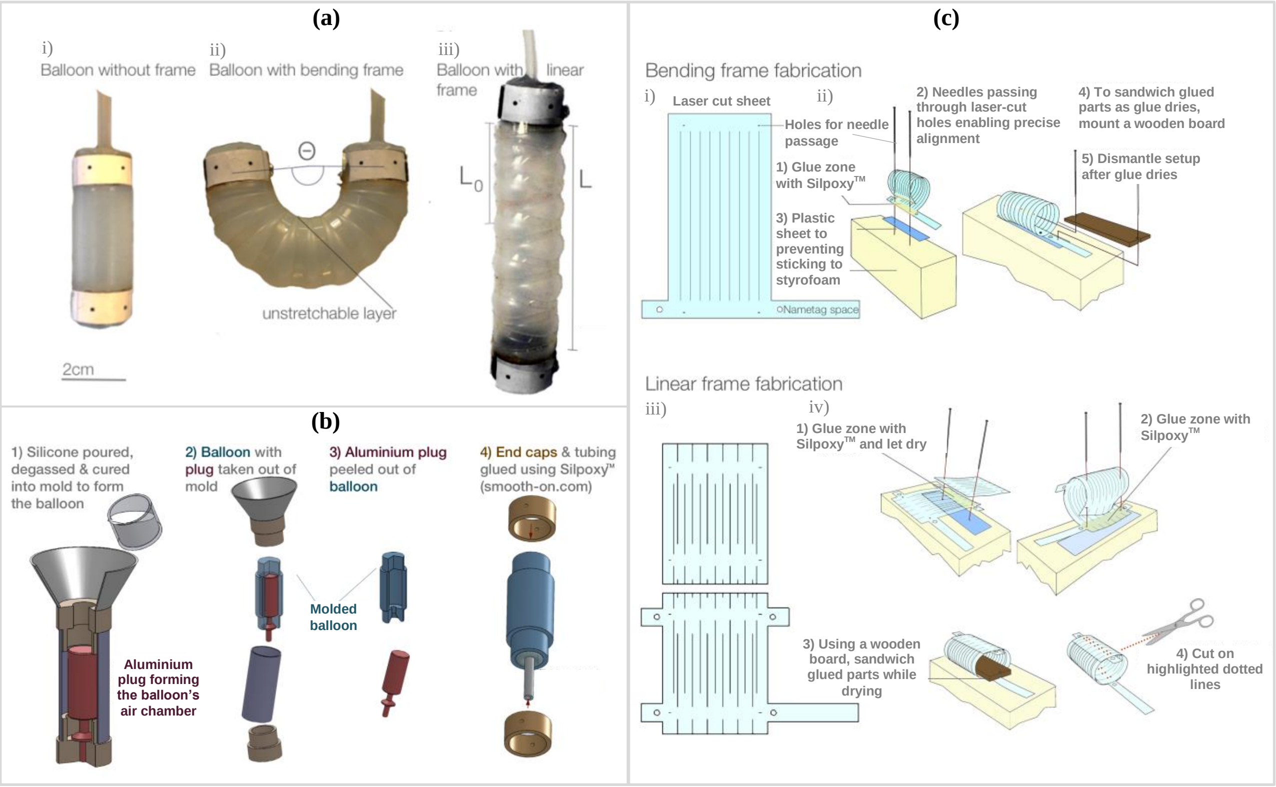

Figure S2: (a) Images showing the (i) stretchable balloon made from silicone elastomer with a shell frame structure attached for creating a (ii) bending and a (iii) linear actuator. The notations for the bending angle Ɵ obtained upon pressurization of bending actuators, and the initial length L_o along with the final length L obtained upon pressurization for linear actuators are defined as shown here. (b) Schematic showing single-step molding process for fabrication of the soft pneumatic actuator body. The actuator body is fabricated from a highly stretchable silicone material, Ecoflex^{TM} 00-30, and comprises of an air chamber. At each end of the air chamber, an additional length of rubber material is provided to facilitate attachment of end-caps for gripping the actuator during testing. The single-step molding process makes the technique for fabrication more robust, repeatable and manufacturable as compared to the traditional two-step molding process. (c) Schematic showing fabrication process for the unstretchable shell wrapped onto the actuator body, to obtain the desired motion profiles in bending or linear motion. The shell is made of a sheet used as a universal inkjet transparency film (Q-Connect^{TM}). The laser patterns created on the sheet define the type and range of motion obtained when this structure is assembled onto the actuator body.

gripping on the active chamber portion can potentially constrict the air flow passage and alter the performance of the actuator. The un-stretchable shell mounted on top of the actuator body surface constrains the actuator body to inflate in only the desired configuration. To grip the actuator for testing, end-caps are attached at the actuator at the border of its air chamber, thus ensuring maximum force/torque delivery. To prevent the shell from slipping out over the actuator body at high pressures, the shell and the body are maintained in place by screws drilled through the end caps and the actuator wall thickness. To track the position of the actuator with a high speed camera, tracking points are also glued onto each end-cap.

The pattern created on the shell surface governs the pattern of displacement obtained with the actuator. Although it is possible to achieve other motion profiles by varying the patterns on the shell surface, bending and linear actuators were selected for further study in this work. The steps involved in the fabrication process of the shells for the two types of actuators are shown in Figure S2.c. To construct the shells, 2D patterns are cut out of universal inkjet transparency films made from PET (Q-Connect™) using a laser cutter. Multiple slits are cut out on the shell surface in well-defined patterns. These slits permit the appropriate level of inflation of the contained rubbery material in the desired direction while the remaining uncut portion provides circumferential reinforcement to avoid excessive radial expansion of the actuator body. The cut patterns are then rolled up and glued into a cylindrical shape, to conform to the shape of the encased actuator body. Unlike the shell for bending actuators, which requires only one laser-cut pattern per shell (see Figure S2.c.i), the shell for linear actuators consists of a glued assembly of two symmetric patterns (see Figure S2. c.iii). For these actuators, having two symmetrically layered glue-zone bands ensures the expansion of the actuator in the linear motion desired. With the exception of hand-cutting away the laces after gluing, the fabrication process is similar to the sequence for the shell for bending actuators, as seen from Figures S2.c.ii and S2.c.iv.

Various shell patterns for both the bending and linear actuators were tested to determine the optimal performance of the combined actuator-shell structure. To create different patterns on the shell, the spacing between subsequent slits was varied, giving rise to a different number of slits on the shell surface for a fixed length of the shell. The number of slits on the shell surface is found to influence the actuator performance, to a certain extent. To study the effect of shell geometry on the actuator performance, various patterns for the shell cuts starting from 3 to 25 cuts for the bending actuators, and 13 to 27 cuts for the linear actuators, were tested. In addition, from subsequent experimental testing, it was observed that at the border of the air chambers, the ballooning rate is approximately double than that at other portions along the the rest of the laces. This is due to the fact that the air flow at the edge of the chamber undergoes a dramatic change in cross-sectional area from a very small inlet diameter to a much larger diameter for the air chamber, thereby introducing vortices and turbulent flow in that region, leading to larger instabilities and inflation of the actuator body in that region. To compensate for this added inflation effect at the end of the chamber, slits that are half the width of the slits in all other portions on the shell surface are created at the extremities of the shell pattern. This design improvement is found to help subsantially in achieving a uniform pattern of inflation with the actuators.
